# Posterior Sternoclavicular Dislocation: A Case Report

**DOI:** 10.21980/J8363Q

**Published:** 2021-01-15

**Authors:** Stephanie Songey, Christopher Goodwill, Kimberly Sokol

**Affiliations:** * Kaweah Delta Medical Center, Department of Emergency Medicine, Visalia, CA

## Abstract

Posterior sternoclavicular dislocations are very rare and pose immediate threat to mediastinal structures should a patient sustain this type of injury. This case report discusses the presentation and diagnosis of a young male who presented with isolated right arm swelling after blunt trauma one day prior while participating in a bull riding rodeo event. He was diagnosed with posterior right sternoclavicular joint dislocation with compression of the left brachiocephalic vein that was seen on computed tomography (CT) angiogram study. Patient was transferred to a tertiary care center for a higher level of care and definitive reduction management. Any history of blunt shoulder trauma or direct sternoclavicular trauma with symptoms to suggest compression of mediastinal structures should make one suspicious for posterior sternoclavicular dislocation. Evaluation is best made by computed tomography (CT) scan. Reduction should be performed by an orthopedic surgeon in the operating room with cardiothoracic surgery available for any vascular compromise that may occur.

**Topics:**

Sternoclavicular joint dislocations, trauma, orthopedics.

**Figure:**
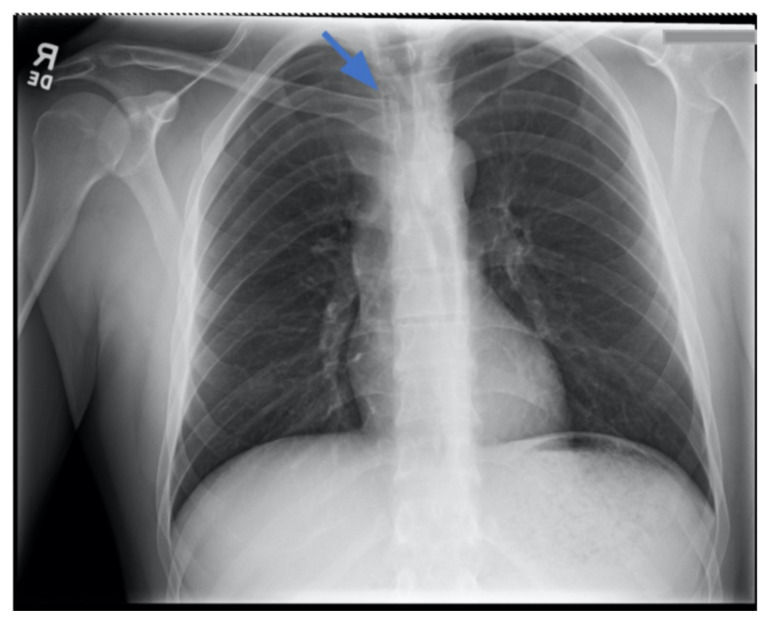

**Figure:**
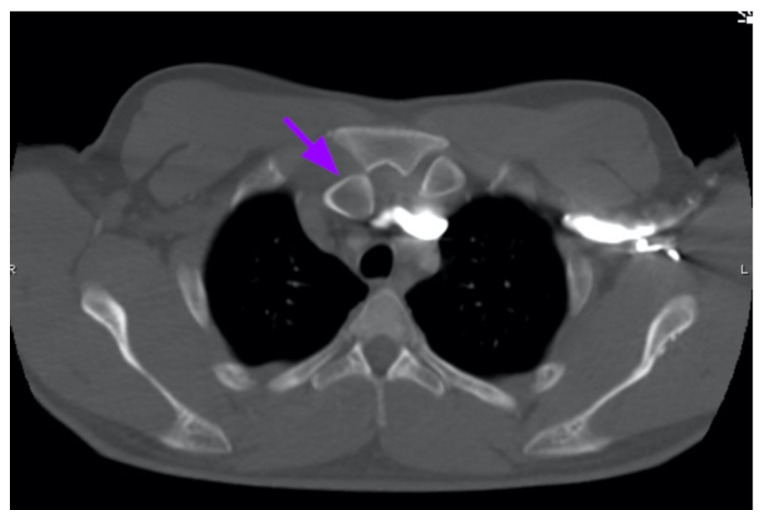

## Brief introduction

Posterior sternoclavicular dislocations are a rare but potentially life-threatening injury. Sternoclavicular dislocations account for 3% of shoulder girdle injuries and of those approximately 5% are posterior dislocations.^1^,^2^,^3^ Many of these injuries occur due to blunt trauma to the shoulder or direct trauma to the medial sternoclavicular joint.^1^,^2^ Identifying this injury is crucial due to the risk of pneumothorax, compression of the great vessels, trachea, or esophagus, and brachial plexus injuries.^4^ Symptoms may be subtle, and X-rays are often inadequate to make a definitive diagnosis.^9^ This case contributes to the latter being able to identify a rare, but emergent traumatic injury. This case took place in a community emergency department setting, requiring transfer to a tertiary care center for definitive management. Being able to accurately and promptly recognize this injury is important when working in rural or small community settings when transfer to tertiary care centers may be necessary.

## Presenting concerns and clinical findings

A previously healthy 26-year-old male bull rider presented to our emergency department with complaints of right arm pain and swelling after being thrown off of a bull the day prior and landing on his right side. Symptoms had been gradually worsening since fall yesterday. He denies any other pain or injuries. On arrival to the emergency department his vital signs were normal. On exam, he was noted to have diffuse right wrist and forearm swelling, significant tenderness over the right clavicle without a step off or tenting of the skin, and decreased range of motion of the right shoulder secondary to pain, though still with intact sensation and pulses. The remainder of his exam was normal.

## Significant findings

Chest X-ray revealed an inferiorly displaced right clavicle at the right sternoclavicular joint (blue arrow). A computed tomography angiogram (CTA) of the chest was therefore obtained and revealed a right posterior sternoclavicular dislocation with resultant compression of the left brachiocephalic vein (purple arrow). Even though the right clavicle is displaced, the anatomy of the brachiocephalic vein is such that it is positioned to the right of midline, placing the left brachiocephalic vein posterior to the right clavicle. The right brachiocephalic and common carotid artery were normal in appearance. The CTA also revealed a comminuted fracture of the left anterior second rib at the costochondral junction that had not been previously seen on the x-ray.

## Patient course

Patient was treated with pain medications and placed in a sling. Patient was ultimately transferred to a tertiary care center for definitive management given the complex nature of the dislocation. Follow-up and long-term outcomes were not monitored by our emergency department team and attempts to follow up with patient were unsuccessful.

## Discussion

Sternoclavicular joint dislocations are rare, accounting for only 2–3% of all shoulder girdle dislocations.^1^,^2^ Dislocations are described as the position of the medial clavicle in relation to the sternum.^3^ The majority of sternoclavicular joint dislocations are anterior dislocations, occurring at a frequency of two to 20 times that of posterior dislocations.^2^ Sternoclavicular injuries are most often secondary to indirect trauma from trauma to the shoulder, or, less commonly, due to direct impact to the sternoclavicular joint.^1^,^2^ Most sternoclavicular injuries are secondary to sporting injuries or motor vehicle collisions.^3^ In our research, there have been no reported cases of posterior sternoclavicular dislocations from a bull riding injury or rodeo event. Rodeos have a long history, and modern rodeo events date back to the 1880s.^10^ A combination of high speed and large bodies of mass in motion create high kinetic energy and high potential for serious injury.^11^ The injury rate of bull riders was found to be 1440 injuries per 1000 exposure hours and the rate of bull-riding injury was 10.3 times the rate of injury in American football, 13.3 times that in ice hockey, and 1.56 times that in boxing.^10^ Bull riding injuries are two times higher when compared to other major rodeo events.^10^ Statistics compiled from 1,939 rodeos over a 34 year period showed 49.8% of injuries occurred in bull riding.^11^ Head and/or face, knee and shoulder injuries were the most common. 10,11 Posterior sternoclavicular joint dislocations need emergent evaluation because they can cause life-threatening damage to mediastinal structures. Patients may present with complaints of dyspnea or hoarseness if tracheal compression occurs and dysphagia with esophageal compression. Arm pain, swelling, and paresthesias can occur with compression of vascular structures and/or brachial plexus.^4^ Some patients may even present with myocardial conduction abnormalities.^5^ Evaluation for sternoclavicular dislocations are very difficult to assess using x-rays, which typically have a very poor sensitivity.^6^ Evaluation is best made via computed tomography with angiography because vascular injury is likely should this diagnosis be made.^7^ While it is not the diagnostic study of choice over CTA, ultrasound has been shown to be able to identify dislocation of the sternoclavicular joint by identification of a widened and irregular sternoclavicular joint space when compared to contralateral side.^7^ If only plain radiography is possible for whatever reason, the “serendipity” view (40 degree cephalic angle) may be helpful to assess for asymmetry of the clavicles.^6^ Treatment options include closed reduction or open reduction with internal fixation. Reduction of posterior sternoclavicular dislocations should be performed by an orthopedic surgeon in the operating room with cardiothoracic surgery immediately available for any vascular compromise that may occur. If closed reduction is unsuccessful, the patient presents greater than 48 hours after dislocation, and/or epiphyseal fracture is found on imaging, open reduction with internal fixation may be necessary.^1^ Post-reduction, patient should be placed in a figure-of-eight brace for 4 to 6 weeks to promote ligamentous healing.^8^,^9^

## Supplementary Information








