# Supplementary figures and images for: Posterior Sternoclavicular Dislocation: A Case Report
Source: J Educ Teach Emerg Med. 2021 Jan 15;6(1):V23–5. doi: 10.21980/J8363Q (PMC10332753; doi:10.21980/J8363Q)

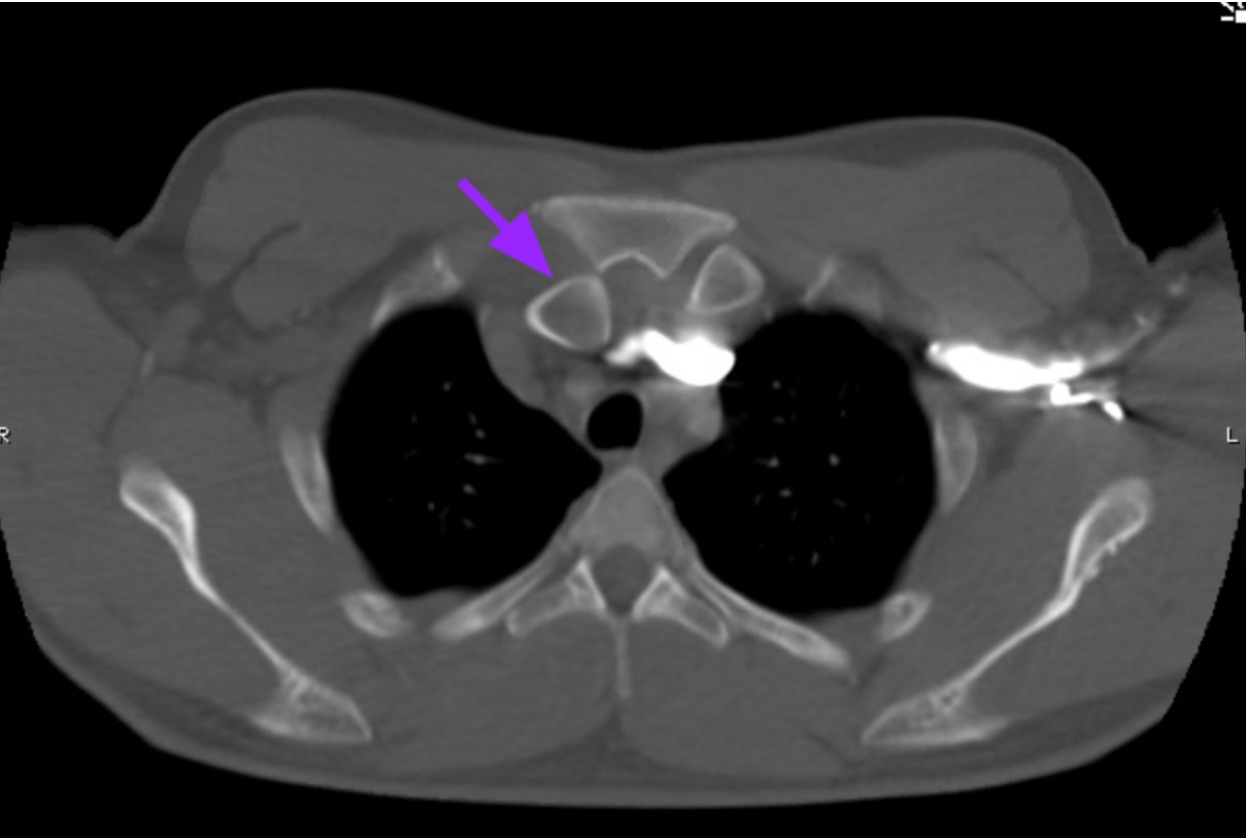

Supplement: Supplementary file 1 [file jetem-6-1-v23-supp1.jpg]

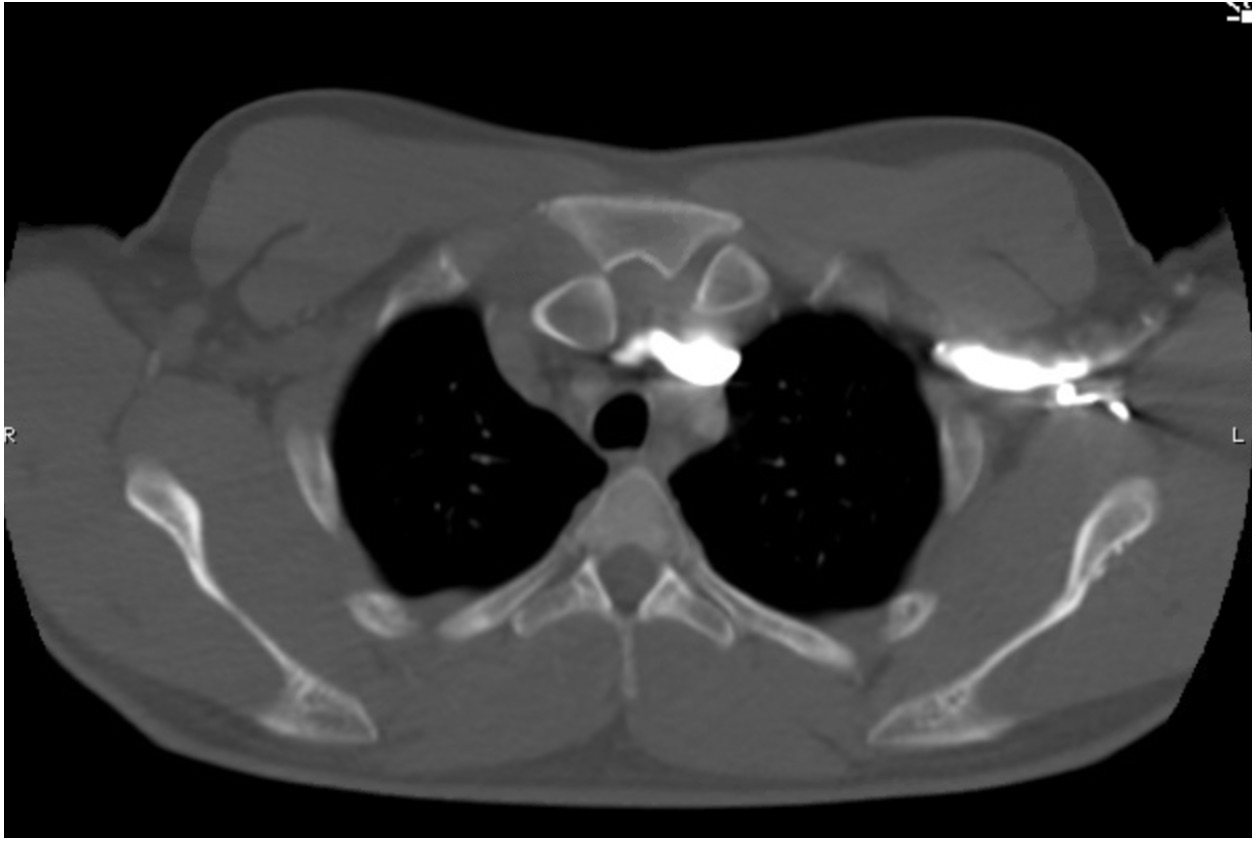

Supplement: Supplementary file 2 [file jetem-6-1-v23-supp2.jpg]

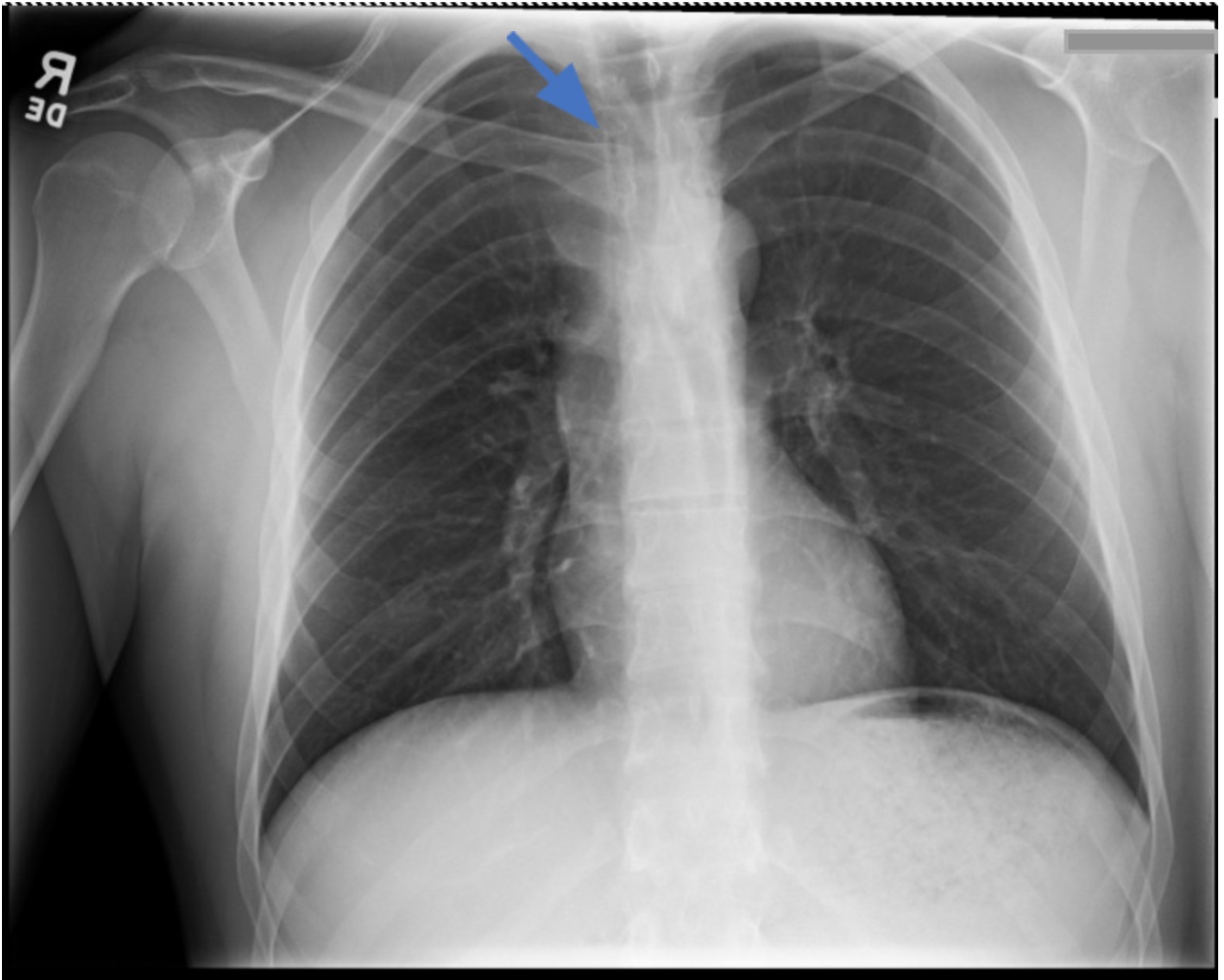

Supplement: Supplementary file 3 [file jetem-6-1-v23-supp3.jpg]

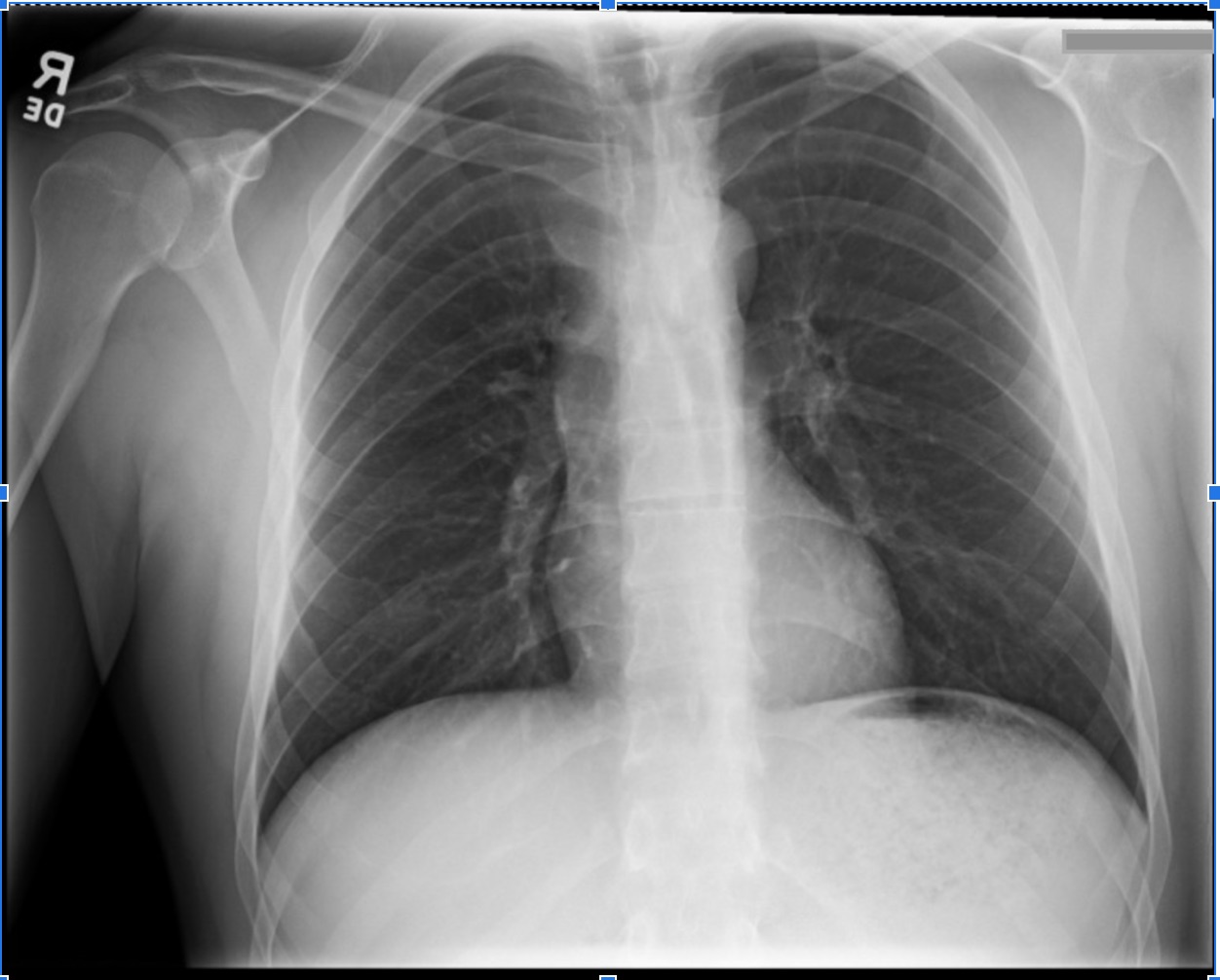

Supplement: Supplementary file 4 [file jetem-6-1-v23-supp4.jpg]
